# "The Hospital" Nursing Mirror

**Published:** 1901-06-22

**Authors:** 


"The Hospital" Nursing Mirror.

Being the Nursing Section of "The Hospital."

[Contributions for this Section of "The Hospital" should be addressed to the Editor, "The Hospital" Nursing Mirror, 28 & 29 Southampton Street Strand, London, W.C.]

### Notes on News from the Nursing World.

### THE WOMEN'S MEMORIAL TO QUEEN VICTORIA.

The Lady Mayoress of Manchester and the Mayoress of Haslingden are among the ladies in the North of England who have issued a circular letter drawing attention to the appeal on behalf of the Women's Memorial to Queen Victoria. At Haslingden a public meeting has been held, and a committee for organising the collection on behalf of the fund for the benefit of the Jubilee Institute for Nurses has been formed; but it has judiciously been decided not to start until after August, in order not to interfere with the usual collections at this time of the year of subscriptions towards the maintenance of the two nurses in the town. The central committee, we are sure, do not wish that any existing organisation employing fully trained nurses should suffer financially from the prosecution of the movement for a singularly suitable memorial to the late Sovereign.

### THE RECOGNITION BADGE AT THE NURSES' INTERNATIONAL CONGRESS.

At a meeting of the Nurses' International Congress Committee held in Buffalo on May 16th and 17th it was announced that the Mayor of Buffalo will open the Congress with an address of welcome. The Congress will also be welcomed by Mrs. Townsend, President of the Women's Union, in whose hall the meetings will be held. All visitors from across the seas arriving in New York or Philadelphia will be met and entertained by the superintendents and nurses of those cities, unless they prefer to make their own arrangements. Delegates and visitors should write to Miss Walker, Pennsylvania Hospital, Philadelphia, or Miss Sutliffe, New York Hospital, West 16th Street, stating the steamer on which they expect to arrive. The "recognition badge" is to be a bow of red ribbon (ribbon to be one inch wide) embroidered or stamped with the name of the country or the association represented by the visitor or delegate. The badges will be worn at all railway stations or steamship wharves by the reception committees of the various cities, and the committee request that they be worn on the left shoulder. Miss Damer, 55 West Mohawk Street, Buffalo, member of the Buffalo Nurses' Association, Chairman of the Local Arrangements Committee, should be written to by intending visitors, stating the sort of accommodation desired, as soon as possible. Prices are likely to be high. A biographical sketch of delegates and other prominent women attending the Congress will be published in a special "Congress number" of the *American Journal of Nursing*. The Press Committee urgently request that delegates send their photographs and a short sketch of their career to the editor, or to Miss Dock, 265 Henry Street, New York, Chairman Press Committee, as speedily as possible.

### SYDNEY HOSPITAL NURSES.

The matron of Prince Alfred Hospital, Sydney, has been interviewed respecting current allegations that the probationers in the Sydney hospitals are overworked. She admitted that a nurse's life is a hard one, but said that it is not too hard for those who have the physical strength for a nurse's calling, and that she tried to take only that class in as probationers. Asked whether the probationers do a great deal of unnecessary cleaning and scrubbing, she replied: "There is some scrubbing, such as keeping the kitchens attached to each ward thoroughly clean, scrubbing the lavatories and the tables in the wards, and there is the cleansing of the ward utensils and the crockery, &c., used for the meals." But she denied that the probationers scrub out the wards, and quietly disproved a preposterous story that a single probationer had "to clean for 120 patients and get breakfast for a similar number." With great long-suffering, she allowed several nurses to be individually questioned, and, generally speaking, they had little fault to find. Similar inquiries addressed to the matron of the Sydney Hospital elicited the information that the probationers in that institution have to do sweeping and dusting and bed-making, but not scrubbing. In the course of her remarks the matron stated that some persons attempted to be nurses who have a very hazy idea of their duties, and are very indignant when they find that things are not entirely to their liking. The nurses' hours at the Royal Alfred Hospital are from 6 a.m. to 8.30 p.m., with three hours off duty each day.

### THE NEW MATRON OF CUMBERLAND INFIRMARY.

Among the appointments announced in our issue of to-day is that of Miss Elizabeth Hodges to the post of matron of Cumberland Infirmary, Carlisle. We congratulate the Governors upon the admirable choice they have made. Miss Hodges has for the last twelve months been assistant matron at Guy's Hospital, having previously filled various important posts in the same institution, and while her services have been greatly appreciated, she has always enjoyed the esteem of the staff. The best wishes of all at Guy's will accompany her to her new sphere of duty in the north. The vacancy at Carlisle was caused by the ill-health of Miss Fawcett, who was appointed in February, 1898. Miss Fawcett worked with untiring energy and earnest zeal in the interests of the infirmary, and her resignation was received by the committee with much regret. At a subsequent meeting of the Governors a resolution was unanimously passed acknowledging the valuable services rendered to the infirmary by Miss Fawcett, expressing their regret at her resignation, and their good wishes for her recovery.

### HALF A SCORE OF NURSES NEEDED IN BRITISH COLUMBIA.

Last week we mentioned that nurses were wanted in some parts of Cape Colony. We now learn that there is an opening at the other side of the world in British Columbia. A correspondent writing from Victoria, the capital, says:—"There is plenty of work here for ten more nurses, and if any nurse would like to correspond with me please give her my address, and I shall be most happy to furnish full particulars." Letters forwarded to us will be sent on to our correspondent.

### GIFT BY A FORMER NURSE TO A NURSES' HOME.

An important feature of the new David Lewis Northern Hospital at Liverpool, which will shortly be opened for the reception of patients, is the Nurses' Home. There will be accommodation for sixty-six nurses. The building itself is connected on the ground floor only with the hospital, and is entered from the main corridor through a large conservatory. On either side of a corridor running the entire length of the block are the bedrooms and bathrooms, and at the end of the passage is a spacious octagonal sitting-room. This description applies to each of the three floors into which the block is divided. The Home is handsomely furnished throughout. On the second floor is a sitting-room which also combines a library. The furniture and the whole of the books in this room were presented by Mrs. Kerr, a former nurse at the Northern Hospital, where she was trained under Miss Anderson, the present matron.

### THREE YEARS' SERVICE IN NATAL.

The Alexandra Nurse at Pietermaritzburg has just completed her three years of service. They have been busy years, as, apart from her nursing of the women and children, she has also been able to assist in the outdoor patient department at the medical inspection room for men. Since the war began it has been a great matter of thankfulness to her that during the whole period only one death has occurred, that of a little child who was delicate from its birth. It is not that there has not been sickness in the camp, for epidemics of chicken pox, whooping cough, bronchial catarrh, and a very serious one of measles, have occurred. Also the usual trials and discomforts of so many infants teething. But the climate is very good for children and altogether the experiences of the Alexandra nurse have been gratifying and encouraging. Last month she paid a very hurried visit to Durban with one of the women returning invalided. The latter had a very comfortable cabin allotted to her use and her husband was with her. The friends of the soldiers who have died in hospital and been buried in the military cemetery, will like to know that on Easter Day ladies placed beautiful wreaths and flowers on each grave, and that this will be repeated from time to time. In many cases crosses are also being erected, and these will be added to later on.

### THE LEAGUE OF ST. JOHN'S HOUSE NURSES.

The inaugural meeting to form an association of nurses past and present of St. John's House was held at 8 Norfolk Street, Strand, on Saturday last. There was a good attendance. The honorary officers and executive committee were elected, and it was decided that the association be called "The League of St. John's House Nurses." The two main objects of the League are:—(1) That by union we may elevate and strengthen our profession; (2) for mutual help, sympathy, and pleasure. The qualification for membership is the possession of a certificate of three years' training, after examination in a general hospital of not less than 50 beds, but the members, or the executive committee, may within two years from the foundation of the League elect as members any nurses who, although not holding a three years' certificate, have filled posts of responsibility for the House. The honorary officers are the President, Vice-President, Secretary, and Treasurer; and the executive committee consists of ten members. There will be a journal published half-yearly containing a list of members with their addresses, official positions, and such details of their career as would be of interest to the League. On Saturday 60 nurses were enrolled.

### AMENITIES AT LIMERICK.

A new matron has been appointed at Barrington's Hospital, Limerick, in the person of Miss Kate Steen, who was trained at the London Hospital, and has been matron of Kettering and District General Hospital. But the election was unfortunately made the occasion for raising the religious question. For instance, Father Shanahan, in proposing the election of Miss Doorley, who had been acting temporarily as matron, said that if that lady was outvoted at the board there would be "heart-burnings and hot words," significantly adding that 95 per cent. of the people treated in the hospital are Roman Catholics. To this the Bishop of Limerick replied "that there was an understanding that if the resident physician was a Roman Catholic the matron should be a Protestant." It was, doubtless, in accordance with the understanding in question, the present resident physician being a Romanist, that the majority of the managing committee—nine as against six—voted, on a division, for Miss Steen. There was no suggestion by anyone that Miss Doorley was not fully qualified for the position, but Mr. Gough, one of the minority, was so angry at the result of the election that he announced his resignation, "as a protest against the eviction of Miss Doorley." According to Mr. Gough's reasoning, a temporary arrangement must necessarily be made permanent.

### CLOTHING FOR THE TROPICS.

In lecturing at the Livingstone Exhibition on "Clothing for the Tropics" this week Dr. Harford Battersby laid great stress upon the necessity for all residents in hot, and especially in moist and hot climates to wear woollen garments, particularly underclothing, and protested against the idea that "woollen" means flannel of a thick and heavy texture. He emphasised the need of great care being taken by nurses in tropical climates to preserve health in tropical climates, mentioning one district near the Niger where such was the humidity of the air that garments taken off at night, unless put into a tin box, would be too wet to put on next morning. Winds and draughts in these climates were great fever-producers, hence the need of wearing wool both by day and night. Besides sleeping in woollen night-gowns, a thin woollen sleeping-bag should, he urged, be used, tied round the neck, so that it would be impossible, during sleep, unconsciously to kick off the clothes. All dresses should be made as loose as possible. The native men in hot climates recognised the desirability of this, and always wore hanging garments. Dr. Battersby mentioned that he adopted the native male costume at one time when he was in Africa, and found the garments equivalent to trousers measured eight yards round the waist. He did not insist, however, that the waist measurements of the nurses need be so voluminous. The other point which he accentuated was the necessity for pith helmets, with a banana leaf inside for additional coolness.

### THE SUICIDE OF AN ARMY NURSE.

The evidence at the inquest on the body of Miss Eliza Holland, an Army nurse, who committed suicide at a Landport hotel, was very saddening. Miss H. C. Morgan, lady superintendent of the Army nursing staff at Netley Hospital, said that she had known Miss Holland for five years. "Before going to South Africa she was acting superintendent at the Station Hospital. She had been sent back from South Africa in trouble, it having been stated that she was addicted to intemperance and taking drugs. Her status, however, had not been altered, and she herself resigned her position as an Army nurse. She was a very hysterical woman, and was excited when she tendered her resignation." Another explanation of the act is that the mental balance of the unfortunate woman was upset by the strain of her arduous work in South Africa. Her brother's statement at the inquest was that she was invalided home on account of ill-health.

### COTTAGE NURSES.

In his speech at the annual conference of the Affiliated Benefit Nursing Association, Dr. Robert Boxall accurately affirmed that the cottage nurses employed under the auspices of the Association are "not what some people might call ideal nurses." The nature of their training absolutely forbids them being regarded in that light. Miss Broadwood made a statement at the meeting from which it appears that 59 persons were sent into training during the year and placed on the cottage nurses' registry, and that nine new affiliated associations were formed, making altogether 112 local societies. That a supply of cottage nurses on the Holt-Ockley system is better than none at all may be freely admitted. But with regard to the contention of Lord Ancaster, that the work of the Association does not overlap that of any other society, it is obvious that the multiplication of inadequately trained nurses must to some extent interfere with the development of the work of the Jubilee Institute, and check the movement for the employment of "Queen's" nurses in country districts.

### WELL DONE, GILLINGHAM!

An excellent commencement has been made by the Gillingham Nursing Association. At the first annual meeting it was stated that two trained nurses from the Queen Victoria's Jubilee Institute, with which the Association has been affiliated, started work on October 1st, and that in seven months they had nursed 160 cases and paid 3,199 visits. On account of the size of the parish and the great distance the nurses have to travel, sometimes twice in one day, bicycles are necessary; and while one of the nurses has been provided with a machine, the other, who possessed one of her own, has it kept in repair at the expense of the Association. The most satisfactory feature of the financial support given to the movement is that upwards of £21 was raised in the dockyard by the employés of one firm by means of weekly subscriptions of a farthing and halfpenny. The farthing subscription in the dockyard amounted to over £12. In fact, the little amounts did much to swell the total of £281 obtained, and the result at Gillingham of inducing so many of the working classes to contribute regularly to the Nursing Association should not be overlooked in other towns.

### THE METROPOLITAN ASYLUMS BOARD.

At the fortnightly meeting of the Metropolitan Asylums Board on Saturday, on the recommendation of the Hospitals Committee, it was resolved that, subject to the sanction of the Local Government Board, Miss J. Webb, the assistant matron of the Fountain Hospital, Tooting Graveney, who discharged the duties of matron for a month in the absence of the late matron, Miss Burleigh, be awarded extra remuneration at the rate of half the difference between the salaries of the two officers. At the same meeting plans were passed for the erection of homes for the female attendants at Leavesden and Darenth Asylums. The proposed home at Leavesden Asylum will provide sleeping accommodation for 40 attendants and the assistant matron, together with sitting-room accommodation for three head attendants, and is estimated to cost £9,420. The home at Darenth will possess sleeping accommodation for 42 attendants and two head attendants, with a large recreation room for the ordinary attendants and a sitting-room for the head attendants, and is estimated to cost £10,250.

### A LEGAL QUESTION AT NEWTON ABBOT.

A case of considerable interest to nurses has arisen at Newton Abbot. One of the nurses at the Workhouse Infirmary was unfortunately taken ill, and it was considered advisable to remove her to the Isolation Hospital, the committee of which have sent in a bill to the Guardians for three guineas, fees for her treatment there. The Clerk to the Guardians advises them that they are not liable, notwithstanding the fact that the nurse was sent there by the Master of the Workhouse. The Isolation Hospital Committee claim that the Board are responsible for the action taken by their servant, and that the fee of a guinea a week is only a nominal fee. The Guardians, however, contend that under the Act the patient is the only person from whom the expenses can be recovered, but neither body suggests that she should be asked to pay, and it would obviously be unjust to her if they did. The case is of more importance than the small amount involved would indicate, for the Isolation Hospital Committee might, if the Guardians maintain their present attitude, decline to accept cases of a similar character from the workhouse in future, and thus create a grave difficulty. As the matter stands at present the clerks of the respective bodies are to secure legal advice on the question of liability. It is hinted that if the Newton Abbot Guardians do not pay, the Isolation Hospital Committee may proceed against them, to test the point which has been raised.

### SHORT ITEMS.

On Tuesday afternoon a meeting was held at 11 Downing Street, by invitation of Alice, Countess of Strafford and Lady Lucy Hicks-Beach, for the purpose of interesting the Mayoresses of the London boroughs in the Women's Memorial to Queen Victoria in connection with the "Queen's" Jubilee Nurses. Promises of immediate action in several boroughs were obtained.—Dr. Robert Lee will lecture on "The Science of Midwifery" at the Midwives' Institute, 12 Buckingham Street, Strand, on Friday, the 28th inst., at 7.45 p.m.—The annual meeting of the Working Ladies' Guild was held on Monday at 48 Belgrave Square. The Guild's work among distressed ladies showed a large increase during the past year; subscriptions and donations were larger. More than £4,000 had been distributed in the past year in the relief of distress and in payments for work.—A garden fête and sale of work done by the children in the home will be held at the Hospital and Home for Incurable Children, 2 Maida Vale, W. The sale is under the patronage of the Duchess of Connaught, and will be opened at 3 p.m. by the Duchess of Marlborough.—The tenth annual meeting of the Invalid Children's Convalescent Nursing Home, Wray Crescent, Tollington Park, was held on Monday afternoon at the University Hall, Gordon Square, Lieut.-Col. Montefiore presiding. The report which was presented states that during the last ten years 402 children had been received, and that in the past year 53 children were admitted and otherwise helped.—On Wednesday afternoon a concert was given at Stratford House, lent by Mr. and Mrs. Murray Guthrie, in aid of the Anglo-American Nursing Home in Rome.

### Lectures to Nurses on Medicine and Medical Nursing.

By NORMAN DALTON, M.D. London, F.R.C.P., Physician to King's College Hospital.

### LECTURE XIII.—THE NURSING OF HEART DISEASE.

(*Continued.*)

In some cases of severe mitral disease the constant shortness of breath may become suddenly increased, so that the patient gasps for breath and becomes blue, while the pulse cannot be felt at the wrist. Death may occur rapidly, or the paroxysm may pass off in a few minutes or more. These attacks are sometimes due to embolism in the lungs. They occur most often at night when the nurse is alone with her patient, and, while sending at once for the doctor, she must administer brandy, and if any cylinders of oxygen be in the house (as they often are in these cases) this is the time when the inhalation of the gas is likely to do most good.

In mitral disease the temperature must be taken at any time if the patient should feel hot, as any rise of however short duration is important. The thermometer should be placed in the axilla, as its presence in the mouth may embarrass the breathing. Another point to note is whether the cough is attended with expectoration or not. The sputum should always be noticed and any trace of blood in it reported.

A nurse should know something about the immediate objects of the doctor's treatment, because in private nursing she may have to report on its effects. The treatment of mitral disease has two objects. The one is to strengthen the heart itself and the other is to diminish the amount of blood which is being dammed up in the veins. When the valvular defect is compensated, general tonics, such as iron, are sufficient. When compensation fails, special drugs, such as digitalis, strophanthus, &c., are given. These drugs actually increase the force of the heart's contractions and also make them less frequent; and, when the physician can only see his patient once a day, a nurse can, by counting the pulse and noting its strength at different times of the day, give a useful report as to the action of the medicine. As regards the second line of treatment, it is found that by giving diuretics, that is, drugs which increase the amount of urine, and by producing purgation, the quantity of fluid in the over-distended veins can be temporarily reduced with great advantage to the patient. In particular the dropsy clears off. Now digitalis itself is a diuretic, but it is usually given with drugs such as acetate of potash, scoparia, &c., which cause diuresis without acting on the heart; so whenever these drugs are being given, the urine, instead of being scanty and dark as it is in mitral disease, should become abundant and pale; and if this do not occur, the fact should be reported. Again, purgatives are given not merely to open the bowels, but to cause large and very fluid motions. Purgatives like jalap, scammony, &c., have this effect, and consequently they are selected for mitral cases. Nurses must remember that to report the mere number of motions per day is not sufficient. If the motions are not large and watery the purgative in use is doing no good and must be changed for another. Other methods of reducing the fluid of the blood in the veins are to incise the dropsical legs or tap the peritoneum. Sometimes the patient is bled. Brandy and ether, &c., are given just to keep the heart going. They whip it up, as it were, and are therefore useful to enable the heart to tide over the worst strain.

Patients with mitral disease may die from suffocation due to dropsy of the lungs, or more suddenly from a large embolus in the lungs or in the brain, or from sudden over-distention of the right ventricle. But the end is often hastened by inflammation of the dropsical parts, such as pneumonia in the dropsical lungs, erysipelas in the legs, &c.

*Symptoms of Disease of the Aortic Valves.*—If the valvular disease be compensated, that is if the left ventricle is big enough and strong enough to drive enough arterial blood all over the body in spite of the leak in the valve, there may be no symptoms. The physician can recognise the defect of the valve by the sounds which he hears with the stethoscope and by the pulse. The characteristic pulse of aortic regurgitation is called the "water-hammer" pulse, and it is a full but collapsing pulse; that is, as the beat occurs the artery becomes suddenly larger than a healthy artery should do (because the large left ventricle puts more blood into it than normal), but, instead of getting smaller gradually, it suddenly collapses, or drops under the finger to the size it had before the beat. This sudden collapse is due to the fact that the blood drops back into the left ventricle because the aortic valves do not shut properly. In compensated cases the big left ventricle beats strongly against the chest wall, but does not cause unpleasant sensations. When compensation fails the heart beats not only forcibly but rapidly, and causes a distressing sensation of palpitation. There is also some shortness of breath. The arterial supply all over the body is insufficient and jerky, consequently the lips are pale, and there may be serious attacks of fainting, caused by anæmia of the brain. At first these symptoms may only occur on exertion. In a simple case no "back pressure" symptoms occur, because the mitral valve prevents the blood accumulating in the venous system, but in many cases the left ventricle becomes so dilated that the mitral orifice becomes stretched, and thus the valves cannot close the orifice, and mitral regurgitation begins to occur, whereupon dropsy sets in, and all the other symptoms of mitral regurgitation. The pulse also becomes smaller, like a mitral pulse, but retains its collapsing character.

In aortic regurgitation the arteries, particularly the brachial, are often "locomotor"—that is, at each beat the artery can be seen to move from side to side under the skin. Patients with aortic regurgitation may die suddenly, especially on some violent exertion; or they may die, also suddenly, of embolism in the brain; or they may die, when the mitral valve gives out, of dropsy, &c., as in mitral regurgitation.

Aortic disease requires only ordinary care on the part of the nurse. In particular sudden strong movements on the part of the patient must be prevented, and brandy must always be on hand in case a fainting fit should occur. If mitral disease should set in, the nursing appropriate for that condition will be needed. Cases of aortic disease are often complicated by "angina pectoris." This disease consists in a sudden attack of terrible pain over the heart, extending down the left arm. There is a feeling of constriction round the chest, the face is pale and covered by cold sweat, and the patient looks alarmed and thinks that he must die. The pain is not worse on pressure over the heart, or on breathing or coughing. The pulse may be weak or irregular. The pain comes and goes in paroxysms, and as it can be relieved by suitable drugs, nurses should be able to recognise it when it occurs and to report the symptoms correctly. It is very rare for patients to die in the first attack.

### To Nurses.

**WE invite contributions from any of our readers, and shall be glad to pay for "Notes on News from the Nursing World," or for articles describing nursing experiences, or dealing with any nursing question from an original point of view. The minimum payment for contributions is 5s., but we welcome interesting contributions of a column, or a page, in length. It may be added that notices of entertainments, presentations, and deaths are not paid for, but, of course, we are always glad to receive them. All rejected manuscripts are returned in due course, and all payments for manuscripts used are made as early as possible after the beginning of each quarter.**

### The Nursing of Lupus Patients at the London Hospital.

By a Correspondent.

### A CHAT WITH ONE OF THE SISTERS.

The recent announcement of the endowment of another lamp for the cure of lupus at the London Hospital has given a fresh impetus to the endeavour to cope with the ravages of that dreadful disease. With the courteous permission of the matron, I was afforded the opportunity of seeing the lamps in operation, and of having a chat with the sister who went to Denmark to learn all about the Finsen method.

As the sister was on duty at the time of my visit, I was conducted at once to the room devoted to the light treatment. As we entered the little yard, I noticed a succession of small tables in regular rows, and inquiring of the nurse who acted as my cicerone for what purpose they were intended, she told me that when the sun was very strong the patients were treated by sun rays instead of the lamps being employed. When we entered the large airy building in which the treatment was proceeding the doors and windows were all open; the fresh air and the warm sunshine kept the atmosphere clear and pleasant. Some beautiful flowers, notably big bunches of roses, were upon a large table, and opposite were some fifteen or sixteen washing basins fitted into a long stand. Of the three lamps that nearest the door was given by Queen Alexandra, and the further one, the "Mary," by Mr. Alfred Harmsworth. They are suspended from the roof, and resemble somewhat the hanging lamps often seen over dining tables in private houses, except that they are gigantic in size. A red frill softens the brilliancy of the light, without in any way detracting from the strength which reaches the patient, for it is conveyed to them through a sort of telescope. Four elevated couches are placed around each lamp; upon each of them lies a patient, whilst a nurse seated on a very high stool—like those used in offices —holds a round piece of glass upon the lupus spot to be treated, allowing the full glare from the telescopic instrument to pour down upon it.

The nurses were all dressed in large holland overalls, made with a deep yoke and buttoned behind, their arms were bare to the elbows, and each wore a large pair of black glasses, which were frequently placed rather low down on the nose, giving a somewhat comical appearance.

### The Visit to Copenhagen.

By the time I had made my inspection, the sister whom I had come to see had a few minutes to spare, so I seized my opportunity and inquired, "Does this room at all resemble the one at Copenhagen?"

"Almost exactly," was the reply. "We copied it as nearly as possible; only here we have three lamps, while at Copenhagen they have five."

"There is no special hospital there for the treatment of lupus?"

"No; only a particular department of a general hospital, as it is here."

"Did you go alone to Denmark—and how did you manage about the language?"

"Miss Morgan, the assistant matron, went with me, but neither of us knew Danish, of course. A good many with whom we came in contact knew a little English, but it was often so rudimentary that it was difficult to obtain all the information we wanted. But, fortunately, I know German, and that language proved a much better medium for conversation, especially with regard to technical words. Nearly all the doctors seemed fair German scholars. Everyone was most kind to us and gave us every facility to study the subject thoroughly."

### Dr. Finsen.

"Did you see Dr. Finsen himself?"

"Oh, yes, and found him such a delightful man! Perhaps you do not know that he is an invalid, or almost so, and can actively practise his profession very little, so he devotes his time and attention largely to scientific questions. I dare say you would like to see his photo."

And the sister took me into a small ante-room where, over the mantelpiece, there hung a framed and signed photograph of a man with a frank, open face, and a kindly, as well as a clever, expression. If it had not been for the dark hair standing up off his face he might easily have passed for an Englishman.

### The New French Lamp.

"Whilst we are here," continued the sister, "you can, if you wish, inspect our last new lamp. It is invented by a Frenchman, made, I believe, at Lyons, and is much simpler in construction than the Finsen lamp."

Here she drew my attention to a copper half cylinder about 13 or 14 inches high standing upon a small table. Near the top was a large glass eye through which as soon as the electricity was switched on streamed the light. The sufferer was put in a chair in a sitting position, and instead of the nurse holding a glass on the spot as in the Finsen method, she pressed the patient's face firmly against the instrument itself. Once, when she inquired if all went well, the patient complained that the spot felt rather hot, and the nurse bade her then press the harder and she would lose the sensation of heat. The sister explained to me that the temperature of the cylinder, as well as that of the glass, was kept down by a stream of cold water which was continually flowing through.

### Its Advantages.

"Has this lamp been long in use?" I asked.

"About ten days; and if successful we anticipate great things from it. In the first place it only costs £1 instead of £120 to manufacture; and though, of course, the larger lamp treats four patients at once, four of these would naturally only cost £4. Then the strength of the electric power required is of course much less when the skin, except for the medium of the glass, comes directly in contact with the light, than when it is applied at a distance of about half a yard. The French instrument can be fitted easily into an ordinary doctor's consulting room: it occupies so little space; and, lastly—and this is a most important matter—a spot four times as large can be treated in one quarter the time."

"How long is the exposure to the Finsen lamp?"

"One hour every day; and the process, to be successful, has to be continued four or five months and sometimes longer; so that you can understand it is a tedious cure, and already enough patients have been promised treatment in the future to last two years."

"How many patients can you treat in a day?"

"About sixty now; but when first we started we began with eight."

"When was that?"

"The 29th of May last year, and we had only a single lamp. We are now just beginning to see the fruits of our labours, as it were."

### The Nursing and Patients.

"How many nurses are employed?"

"Thirteen, and two sisters. To the outsider the work of holding a glass in position, though tiring, looks very simple, but it demands far more care than you would imagine. If the glass does not touch the skin, it acts as a burning glass, and may produce a sore at once, and practically the nurse has to keep her attention keenly fixed on her patient all the time. The nurses get interested in seeing the gradual improvement in a disease once considered incurable, and as a class we all agree that lupus patients are some of the very nicest. It is quite wonderful to see the change which comes over them in a few months when they begin to realise that it is possible they may be able once more to mix with the world. The look of leaden despair, or resigned hopelessness, gradually disappears, and light and happiness return once more to the face. They are all photographed before the cure is started, and frequently afterwards when we show them the photos, they will hardly believe they were ever so dreadful to gaze upon."

"How long do the nurses work?"

"From 8.30 A.M. till 5 P.M., with an interval for dinner. Each nurse takes five patients in a day."

"Are these all out-patients?"

"Except about two who are too destitute, or live too far away to be sent home."

"I notice that all have the disease in the face except one girl, who is being treated for lupus in the arm."

"Preference is always given to face cases. The disease appears to be more common amongst women than men."

"Does the light ever cause serious injury?"

"Not if due care be taken. Some skins are more sensitive than others, and are affected more easily. Every spot, after treatment, is dressed by a nurse with simple ointment before the patient departs."

THE RISK OF CONTAGION.

"Generally speaking, does the exposure to the light produce pain?"

"Sometimes to a slight degree at the time, and the next day there is a certain amount of inflammation and soreness. But that is all."

"Lastly, do the nurses run much risk of contracting the disease in ministering to the patients?"

"Very little, and every precaution is taken. Each nurse at work wears a big holland overall, which she takes off before leaving the room, and keeps her arms bare to the elbow, so that they can be easily washed. Each has also her own basin in which she washes her hands, and it is one of the rules that she must do so between each case as well as at the end of the work."

Then, as sister's off-duty time had come, I waited till she had performed her ablutions, and we walked through the gardens together.

### Examination Questions for Nurses.

### LEWISHAM INFIRMARY.

THE following are the examination papers which were given out on Friday, June 7th, to the 1st and 3rd year nurses at Lewisham Infirmary:—

EXAMINATION FOR 1st YEAR PROBATIONERS.
TWO HOURS.

Questions 1 and 2 must be attempted, and either 3 or 4.

1.—Describe the Bony Pelvis.

2.—Describe the process of digestion in the stomach.

3.—Mention the nursing points of any acute medical case you have observed.

4.—What do you understand by the following terms:
1. Hemiplegia. 5. Variola.
2. Paraplegia. 6. Carbuncle.
3. Apoplexy. 7. Peritonitis.
4. Pneumonia. 8. Intertrigo.

FINAL EXAMINATION FOR 3rd YEAR NURSES AT COMPLETION OF TRAINING.
THREE HOURS.

Not more than THREE questions to be attempted in each group.

A.

1.—What changes does the food undergo in the stomach? Describe how milk may be artificially digested.

2.—Describe the differences between the circulation in (*a*) the arteries and (*b*) the veins.

3.—What precautions should be taken in the nursing of infectious cases to prevent the spread of the disease to others?

4.—What special precautions are necessary in the nursing of (*a*) paralysed patients and (*b*) those liable to fits?

5.—Describe the mode of onset of enteric fever. How is this malady acquired?

6.—What is apoplexy? Mention some diseases in which apoplexy may occur as a complication.

B.

1.—Describe, as fully as possible, the atlas and axis.

2.—Mention the precautions you would adopt if a case of erysipelas were to arise in a surgical ward.

3.—Describe the nursing points which would arise in a case of rectal obstruction requiring inguinal colotomy, from its admission to its discharge.

4.—What do you understand by the following terms:
1. Concussion. 4. Epilepsy.
2. Compression. 5. Apoplexy.
3. Acute Alcoholism. 6. Syncope.
7. Coma.

5.—Describe fully the interior and contents of a surgical ward containing 28 beds; noting aspect, material, cubic space, floor space, beds, linen, furniture, and offices.

6.—What is ophthalmia and how would you nurse it?

Dr. Toogood, medical superintendent to the Infirmary, is examiner in each case, and Dr. Rose Bradford, physician in University College Hospital, is associated with him in the examination of the 3rd year nurses.

### CORK STREET HOSPITAL, DUBLIN.

The questions given at Cork Street Hospital, Dublin, this month at the Junior Nurses' Examination, by Dr. J. Marshall Day, are as follows:—

1.—Describe what you see when you look into the open mouth, enumerating the structures shown.

2.—Give the proper method for disinfecting a room.

3.—How do you make white drink, sweet whey, whey?

4.—How do you peptonise with liquor pancreaticus?

5.—How much sleep does a patient require? How do you get a patient to sleep?

6.—What do you mean by the terms statim, dyspnœa, pericarditis, haustus, pulvis, ex aqua (dorsal-decubitus)?

7.—How would you arrange a room for a fever patient in a private house?

8.—How do you pack a patient?

The Senior Nurses' Examination Paper, also set by Dr. Day, was as follows:—

1.—Give the symptoms of perforation in enteric fever.

2.—What would you do in a case of hæmoptysis until the doctor arrived?

3.—How would you stop the bleeding from a varicose ulcer?

4.—Explain fully the method you would advise for making beef-tea in a private house.

5.—How would you prepare a patient, the instruments, and the room for an operation? How do you sterilise the dressings, &c.?

6.—What symptoms point to acute renal disease in scarlatina?

### Suggested Reforms in Military Nursing.

By an Army Sister in South Africa.

(*Concluded from page* 147.)

### Proposed Changes.

There seems, at any rate, to be required, (1) a six months probation, longer for men than women nurses, as the former are less adaptable and slower at grasping new ideas and methods; also because orderlies are drawn from a less educated class than recruits to the nursing profession. A three months' trial, which might really test a woman, would possibly not give time for a man to rise to the mental standard required in an orderly. (2) A higher rate of pay to attract good material; substantial extra pay to be given for night duty; and sleeping on duty to immediately disqualify a man for such work for a given length of time. (3) The two-hourly shift system to be abolished once and for all. Duty hours to be on civilian lines; these having been universally found to work better for the patients as well as for those in charge of them. (4) Drunkenness to be a final and immediate cause for dismissal at any period of service. (5) The War Office to reserve to itself, as do all boards and committees of civilian hospitals, the right to dismiss any orderly, who after probation should yet prove himself unsuited to nursing, without the commission of some real "crime"—using the word in the lay, rather than in the military acceptation of the term. (6) A systematic course of instruction, with examinations as to proficiency at stated intervals, to be given to orderlies. At present instruction, theoretical and practical, is part of an orderly's training; this is followed by an oral examination consisting of about eight questions—in some instances a temperature-chart has to be made out, and the orderly is asked whether he understands the use of the clinical thermometer. Having passed through this ordeal he can draw "call" money in addition to regimental pay, so that a little inducement is offered to pass. A text-book of nursing is also supplied, but there is no compulsion to read it, and knowledge of it is not necessary to passing the one and only examination. Obviously, therefore, theory on which good intelligent practice is based holds a small place in an orderly's training. The men are usually very glad to learn the reasons for certain lines of treatment, and would probably much appreciate a more complete course of teaching. (7) That on attainment of a certain proficiency, orderlies should be raised in rank and serve in the wards as seniors—corresponding somewhat to the rank of staff nurses. Now, none but privates serve in the wards—being drafted off on promotion to clerk's or wardmaster's duties—the patients losing the advantage of an experienced orderly, or the orderly forfeiting promotion by remaining in ward work. These reforms, inadequate as quite possibly they may be, and not embracing many points to which attention might well be turned, would, however, tend to greatly improve nursing by orderlies, would add to a higher opinion of military hospitals than is prevalent, and would undoubtedly benefit the patients.

### The Question of Discipline.

Under the heading of discipline, reform is called for urgently. Incongruous as it may sound, there is no civilian hospital of my acquaintance, and I have been on the staff of five, where institutional discipline is so thoroughly neglected, I had almost said unknown, as in a military hospital. In those of large size—non-military—the sister is a queen in her ward or wards. She is held responsible for all that goes on in it; has complete and undivided control of whatever concerns her patients, subject of course to the doctor's authority; all matters of management are in her hands alone; the nursing staff under her recognise that as she takes the sole responsibility her word should be law, and give to her tha respect outward and real that such a position demands. This state of things has no counterpart in the military system. A nursing sister is little more than a figure head, and those in authority, at any rate in England, give no credit for—not that it is wanted—but rather discountenance work on the part of a sister who ventures to go beyond the bare letter of regulations or to overstep custom among military sisters by doing more than give out medicines and distribute stimulants. This war has called so many civilian nurses into the field that perhaps such an assertion may be disputed; but if the subject is discussed with an R. A. M. C. Colonel, Major, or Sister Superintendent, they will tell you that the sponging of enterics is a matter for the orderlies; that the making of beds is no part of a sister's duty, even though she be surrounded by incompetent subordinates; that the orderly has charge of the linen, though he does not hesitate to put damp sheets on the beds. The sister has no responsibility for the cleanliness of the ward, which is regarded in a civilian hospital as one of the most onerous duties that fall to her position, since it materially affects the well-being of the patients. This duty is entirely in the wardmaster's hands; any complaints as to dirtiness, slovenliness, must go through him to the orderlies, and they know that she has no power to insist on cleaning in her ward; it is not her department, and they are not responsible to her for it. And as the wardmaster is comparatively an uneducated man, whose home surroundings have perhaps not been of a high sanitary standard, he does not exact, except when an official inspection is imminent, that cleanliness which is part and parcel of a well-managed ward. The comment of a patient in a ward, which was being cleaned and tidied just prior to a visit of a lady of exalted rank, is here very apposite: "Well, sister, it's the only way of getting the hospital clean, when —— is coming to see it."

### The Diet Order.

Again as to diet. The food supplied to soldiers in hospital is, as far as my experience goes, on the whole excellent—certainly above the average obtainable in a civilian institution. It is there in plenty, of good quality (tea being a notable exception, and this probably because it is made in huge quantities instead of each ward or division supplying its own gallon or gallons), and in great variety. There is no stint in stimulants or extras, and though allowances are cut very fine, it is always possible, applying at the right time and through the proper channels, to obtain what is required for the patients. The British soldier is not fastidious as to the serving—his requirements seem to be quite satisfied in this respect. But the ordering of the diets, these having been selected by the doctor, is in the hands of the orderly of each ward, and seems to him such a stupendous task, that frequently other duties are neglected while he pores over blue papers, and, after an infinitude of trouble, evolves a list which is copied on to a printed form and sent to the quartermaster. Next day—a diet short, an extra missing, several ounces of stimulant not supplied, attest the fact that "where there is a will" in an orderly "there isn't always a way." The patient's announcement "it doesn't matter, sister, it will do to-morrow" doesn't satisfy. But if the sister, as some do, takes the precaution to check the diet order, she is overstepping her department, and might quite easily be told as much. She is not responsible for making up the diet lists, though it is part of her duty to see that the patients get what is ordered.

### The Effect of Limited Authority.

This limited authority in the ward has its effect on the relationship between orderly and sister. As was once remarked "the first thing a soldier looks for in a superior is the shoulder strap and stars, his respect being in exact ratio to the number he finds there." I am prepared for contradiction when I say that the majority of orderlies do not behave to the sisters with the respect they should—with not a quarter of the respect that a probationer, who is quite likely her social equal, pays to the sister in a civilian hospital. They will lounge about in a chair and listen to instructions about patients, and are ignorant of the fact that a military sister ranks as a subaltern and should be treated with the same courtesy. Many sisters let this matter slide, either from fear of becoming unpopular by upholding the principle of discipline, or through the paralysing thought that it is such hopeless work to try and get things as they should be. But why trouble about small details? Is it not a petty vain spirit that takes notice of such trifles? says some objector. Not when it is a question of joint work between ignorant, unrefined men and educated ladies; concession is merely construed by them into weakness, and never by anyone more than soldiers, who so well understand what discipline means in other ways. One has happily proved that it is compatible for a sister to maintain discipline and take a kindly interest in her orderlies, and that they should be assured on their part that sister is a friend.

### More Nurses Wanted.

More sisters are required—and more orderlies; the former that they may be able to look into all that concerns the patients better than is now possible, and the latter that work may be done thoroughly and methodically. Perhaps want of method is one of the most striking characteristics of an orderly's nursing. I have frequently seen dusting in full swing before sweeping had commenced—and sweeping started before beds were made. The first thing a day orderly undertakes when he comes on duty in the morning, at any rate in some hospitals, is a smoke, then his own ablutions, and a good half hour is wasted before any work whatever is begun—incredible as this sounds to a nurse. This is of course the consequence of inferior training and of the sister's restricted authority. These and a dozen other things justify, in my opinion, the term "figurehead" being applied to an army sister. I have by no means exhausted the topic of military nursing reform; but these salient points certainly require careful attention.

### TRAVEL NOTES AND QUERIES.

Glasgow Exhibition (L. B.).—You give no pseudonym. No, the tourist ticket will not allow of a break of journey except on specified conditions. They usually last six weeks and two months. You must take a separate ticket to and from Worthing.

Glasgow and Edinburgh (Scotland).—Your best way will be to take a tourist ticket to Edinburgh (£2 12s.) or to St. Andrews (£2 17s.) and go to Glasgow and back to Edinburgh independently: it will cost about 15s. more. But even then it is considerably cheaper than making the round. All lines are the same price. I do not know the cost of tourist tickets on the English lines—I have no list at hand—but you have only to call at your own station and they will give you the correct price. Both lines are the same.

Knokke in Belgium (F. D. Rectory).—We do not reply by letter unless 2s. 6d. is sent for the Convalescent Fund, but I hope this will catch your eye. No lodgings in Knokke—that is not continental style. Apply to the proprietor of the Hôtel de Bruges or the Hôtel de la Marine. A little more expensive but nearer the sea, Grand Hotel de Knokke. Second-class return to Ostend *viâ* Dover £1 16s. 4d.; about 2s. more will cover the rail and tram to Knokke. A cheaper way is by steamer from Tower Bridge, 2nd return 9s. Full particulars of this place in the Nursing Mirror for September 1st, 1900. It will be sent to you on receipt of three stamps.

North Wales (J. H. W.).—We do not reply by letter unless you send 2s. 6d. for the Convalescent Fund, but I hope this will meet your eye. Llanrwst is a very good centre. I cannot tell you of rooms or lodgings. Go to Hotel Victoria for the night and look about. I am not aware of any boarding-houses there yet. Early in July accommodation will be cheaper than in August. It is a truly lovely district. The article you inquire about was in the Mirror for August 26th, 1899. It deals with the district you want to learn about. Penmaenmawr would be rather more expensive: there is a boarding-house there, well spoken of, called "Fernbrook." At Colwyn Bay there are many: try the "Edelweiss" or the "Home from Home."

### The Nurses' Co-operation.

A meeting between the committee and nurses of the Nurses' Co-operation was held on Friday, June 14th, at the Howard de Walden Home. The meeting was largely attended, the room being overcrowded. Mr. Pitts took the chair at the request of the Committee of Management. Miss Geddes moved, and Miss Tonks seconded, "that Miss Morten take the chair"; this was carried unanimously. Mr. Pitts said it was out of order, and refused to leave the chair. Miss Gelston moved as an amendment that Mrs. Large take the chair; she said that as there was friction between the nurses and the Committee of Management, it was only fair that an independent person should be in the chair. A nurse asked why it was in order for Mrs. Large to take the chair and not for Miss Morten. Mr. Pitts sank back again into the chair, and the nurses laughingly allowed him to retain the coveted seat. Mr. Pitts made a lengthy statement about long-past events; the nurses interrupted frequently. He then called on Miss Clutton, who rose with a tremendous typed financial statement. After a few pages the nurses refused to hear any more; they said they had come to the meeting with great difficulty and some expense, and they wished to proceed at once with their agenda, which dealt with certain pressing difficulties, and not with the general affairs of the Co-operation. Mr. Pitts said that it was a special meeting of the nurses and the committee, and that no voting could be allowed. The nurses said they had asked for a general meeting, and they produced the printed announcement of former meetings described as general meetings, and to these both nurses and members had been summoned. They wished to know why their desire to meet the members was frustrated, why letters were ignored, and why things were so persistently delayed. Mr. Pitts said that perhaps, then, the nurses might speak, though not vote, but only the nurses. Miss Gelston, Miss Tonks, Miss Turner, Miss Lawrence, Miss Rogers, Miss Gerhardt, and Miss Thompson then moved the following resolutions, which were all passed by acclamation, since voting was not allowed. There was not one dissentient voice:—

1. That this meeting requests the Committee of Management, the secretary, and the honorary treasurer to resign.
2. That the Committee of Management be asked to request the lady-superintendent to reconsider her resignation.
3. That the articles of association be so altered as to give the nurses a larger representation on all committees.
4. That the Committee of Management be requested to take steps to inform the nurses monthly of all events of importance concerning the welfare of the Co-operation.
5. That all paid posts, save those of staff nurses, be advertised, and that in the annual balance-sheet the salaries of all officers be stated.
6. That these resolutions be sent to our honorary solicitor, with a request that he give them effect.

Mr. Pitts then called on Mrs. Large, who rose with a written speech in her hand. She said the nurses should be loyal to the committee. Miss Morten then demanded to be heard, since it was in order for Mrs. Large to speak. She said that the committee should be loyal to the nurses. Dr. Turney said that the committee were doing their best for the nurses. The nurses asked why the committee delayed their good deeds, and received the assurance that the committee had repented their declaration not to meet the solicitor for three months, and were to see him next week. After further very brief but pointed remarks from the nurses on the question of the treatment meted out to the lady superintendent, and some lengthy and unbusinesslike replies from the committee, a stormy meeting was brought to a peaceful close.

### Echoes from the Outside World.

### AN OPEN LETTER TO A HOSPITAL NURSE.

Not only throughout Russia, but in all parts of the civilised world, great sympathy will be felt with the Czar and his wife in the disappointment they have experienced owing to the arrival of a fourth daughter instead of a son and heir. It was fondly hoped, and much desired, that the little stranger would solve the question of the succession in the way most congenial to the Russian people. At present, of course, the heir is the Czar's surviving brother.

I went on Tuesday to the second Livingstone Exhibition at the Westminster Town Hall. The opening ceremony was performed by Sir G. Taubman-Goldie. The chief features of interest were the relics of the great explorer, including his medicine chest, of a most primitive character; his case of surgical instruments containing an unpleasant looking saw, suggestive of terribly painful operations under untoward circumstances; a section of the tree with inscription from the shores of Lake Bangwenlu, Central Africa, under which the heart of Dr. Livingstone was buried; and his watch and key. I think that Livingstone must have had sad trouble with that key, and spent some time in searching for it, for it is most carefully tied with African grass on to a piece of wood, so as to keep it safe. There was something very pathetic in the last entry in the note-book in which the explorer spoke of being laid low by fever, and close to it I noticed his ink-bottle and pen, which have been carefully preserved. There were photographs at the exhibition by various people taken in all parts of Central Africa, and in a large room beyond the hall every requisite for a traveller, man, woman, or even long-clothes baby, was shown. Of course samples of their wares were distributed by many of the firms, and it was quite amusing to note the curious collection of things which some of the ladies bore away with them. There were a goodly number of nurses present, and they, of course, had many little tins and boxes pressed upon them; but the missionary students were in most cases left alone, because, I suppose, they were only men.

It is only five years since Signor Marconi took out his first patent in the matter of wireless telegraphy, and already the invention has made great strides and been widely adopted. The system is fitted on thirty-seven of the ships of the Royal Navy, and now the first installation has been arranged on board a liner. The *Lucania* left Liverpool on Saturday, and communication was continued with the land until she was fifty odd miles beyond the Irish coast. At her masthead was a small spar two or three yards long, from which hung a wire leading to a small house of wood on deck. Inside this deck-house the batteries were concealed, where the electricity generated on the ship was stored. Not only were greetings exchanged by the Cunard and the Marconi Companies, but any private individual was at liberty either to receive or to send a message, the tariff being sixpence halfpenny a word. The halfpenny is for telegraphic transmission on land, the sixpence the charge by the Marconi Company. There was quite a crowd of messages sent off by the passengers, and a fair number received, notably one from the home of a gentleman who had left his wife dangerously ill to say that she was slightly better. It is maintained by those who ought to know that when the station in Sable Island, off the banks of Newfoundland, is finished there will be only two days during which intercourse with an Atlantic liner will be impossible; also that the more general the system becomes the greater will be the safety of vessels. Thus, if a ship breaks down in mid-Atlantic the time is at hand when she will be able to communicate with invisible steamers miles away and secure their assistance. Also, it will be practicable to send warnings to ships in peril near the shore, a position far more dangerous than being on the boundless ocean. Neither fogs, nor storms, light nor darkness, can influence the electric waves, and they pass over hills or obstacles as easily as if these did not exist. Against all the perfections and other advantages there is one drawback. People who have hitherto taken their holiday on the sea in order to ensure perfect freedom from business worries, will no longer be safe. Signor Marconi's emissaries will in time follow the bishop, the stockbroker, the barrister, and the merchant with worries and wants even to the North Pole, and by the end of the present century there will probably be no single spot on the round earth where a man can retire and feel that he has in very truth cut himself off from his fellows.

One cannot help remarking that at any particularly fashionable function the ladies are still mostly clad in black and white, grey or mauve. Colours are seen a little in the streets, but it is wonderful how many people totally unconnected with the Court in any way have voluntarily chosen to give up any material of brilliant hue, and to confine themselves for the present to half-mourning tints. Asking a "mere man" the other day if he had noticed this adherence to sober tints, he replied, "Yes, and I think I have never seen Englishwomen so well dressed." I was not surprised because I have long grasped the fact that most men like to see their womenkind neatly, rather than gaudily, dressed, and that dark or self-coloured garments gain more praise from them than subtle combinations. Just now there is a sameness about dress at outdoor gatherings, because, whatever else the up-to-date woman may give up, nothing will persuade her to relinquish her boa. It is amusing to notice that on tall women or short women, elegant women, or stumpy women, the inevitable ruffle in chiffon, or tulle, or net, in white or black, or with a careful blending of the two is everywhere *en évidence.* Lace is used on everything—parasols, hats, and capes, silk and muslin, and even light woollen materials. Often a coarse guipure and a fine French black lace, is worked in together, and upon black and white foulards the mixture is especially pretty. Velvet is still much used, not always so very narrow as of yore, and black velvet as a waistband has quite taken the place of petersham or ribbon. Flounces can hardly be too many or too fanciful as a trimming to the skirts, and all hats are raised off the face by a stiff band inside, though its existence is often hidden by twists of velvet or sprays of flowers. Large hats have quite superseded toques, and as for bonnets they are only to be seen upon ladies of 70 years and upwards.

Women are ever keen to originate, if possible, something new, because greater success, as a rule, lies in originality than in any well-worn idea, however admirably it may be carried out. Lately a "score" has been made by a money spender in Paris and by a money maker in England. In the French capital a lady has just invented the "singing dinner." Not a word was breathed when the invitations were sent forth that the dinner would in any way differ from an ordinary entertainment, but before the end of the soup, suddenly a well-known music-hall singer appeared in a bower of flowers, and warbled out a Parisian ditty, sufficiently amusing, but not requiring any serious attention which could detract from the business of eating. During the next course an invisible choir sang "soft and low," so that those who wished to converse could still do so in an undertone, whilst those who preferred to listen were at liberty to please themselves. These two features were repeated at intervals. The genuine "diner-out" who looks upon the ménu as the feature of the evening will rejoice at a chance of stopping his lady's conversation, thus enabling him to devote himself heart and soul to the viands; but, I fancy, most women who enjoy a chat quite as much as a casserole will rather set their faces against "singing dinners." The other novelty is the establishment of a floating tea-shop on the river. A lady, who has probably had some experience in the disasters which often attend the riverside "brewing" of tea with a kettle and spirit lamp, has taken a house-boat between Boulter's Lock and Maidenhead and fitted it up as a dainty tea resort where everything to refresh the inner man and woman can be obtained with comfort and even luxury. When the weather becomes warm enough to make the river the desired haven of weary souls and hot bodies, the new tea palace should prove a great "boom."

### Nursing in a Bavarian Hospital.

BY A CORRESPONDENT.

AMONG the many and varied experiences that befall a British subject abroad, not the least interesting may be reckoned a visit to one of the larger German "Stiften" at Munich.

Perhaps some portion of the interest attaching to "Rothes Kreuz" may be attributable to the fact of its being under direct Royal patronage. To this institution the Crown Prince is visiting surgeon. Here, in the cause of religion and charity, "Königliche Hochheit" lays aside his imperial dignity and devotes himself to the alleviation of human suffering. And now is manifest the power of poverty, for, while a paying patient must content himself with the attentions of an ordinary untitled physician, a penniless one may become the privileged recipient of his future sovereign's skilful ministrations.

A PRINCE PERFORMING AN OPERATION.

I was interested one day in hearing that a poor woman was even then waiting the advent of the Prince to perform a difficult operation, and a bright-faced little nurse explained how unostentatiously this good man goes about his self-imposed labours. Whatever respect his position may demand under other circumstances, at Rothes Kreuz his Highness wishes to be considered a doctor pure and simple.

THE SCHWESTER'S GARB.

It has been remarked that in the average German mind a preference for utility, rather than beauty, prevails. To this rule the Bavarian Schwester's garb proves no exception. She wears a blue, on Sundays a black, gown, and if the English invalid may experience a sense of disappointment at the conspicuous absence of smart cuffs and snowy apron, he will soon become reconciled, and even learn to welcome his homely attendant, with hair parted plainly in the middle like a Dutch doll, and an old-world white head-dress drawn closely round her rosy face.

THE CLASSES OF PATIENTS.

Accommodation at Rothes Kreuz is of three classes, the lowest being that in which house-room and attendance are granted free. A second-class patient may be obliged to share his apartment with another, although this regulation is waived at the discretion of the directress. German beds are something distinctive, provided with a pillow which might have comfortably supported the heads of two Goliaths of Gath, and a "plumeau," or eiderdown, which is heaped upon the patient, who, if she be a lady, submits, among other things, to having her hair plaited into neat little pigtails.

SCHWESTER SWABS THE FLOOR.

Nurses are frequently their own wardmaids, and to the more fastidious English mind there is something not altogether agreeable in having one's ablutions performed by the Schwester who has just been swabbing the floor with a mop and a pail of water. Cleanliness is, however, one of the many commendable features of Bavarian hospital life. Probationers entering this hospital assume, like nuns, names other than their own. The society is unsectarian, and among its ranks both higher and lower Germany are represented. Lutheran services are held in the chapel for such as can or care to attend, while a devoted Herr Pastor visits any who will welcome him to their bedsides. As is usual elsewhere, the Roman Catholic priest attends his own flock. Here virtue must be its own reward, gratuities to nurses being strictly forbidden. Boxes are kept, however, in which grateful patients may deposit what they think fit, and this is afterwards divided among the sisters.

THE INVALID DIET.

Whatever may be said of German cooking in general, their invalid diet leaves much to be desired. Animals' brains, pale and diminutive sausages served standing on end, in a cup of hot water, and various concoctions of a brothy nature, known comprehensively as "suppe," form some of the staple commodities. Ices, an ingredient of which is coarse brown bread, "mehlspeise" (flour pudding), ham and venison may be added to the list. Calf's foot jelly comes up an unappetising grey mash, bread and milk appears as the inevitable soup, but though our benighted sisters further south know not how to poach an egg, and in health and sickness exhibit many manners and customs contrary to English prejudices, they are as a body industrious and whole-hearted in their work.

"MADE IN GERMANY."

To hearth-and-home worshipping John Bull, a mere mention of the Vaterland sometimes acts as would the proverbial red flag upon his namesake, though it is a very ordinary thing to find the enterprising Teuton firmly and economically established behind the counters of our banks and bakeries. Yet this very people, while taking from us with one hand are giving with the other. We, among many more, have reaped the benefit of their numerous discoveries in the art of healing and its sister sciences, and as once upon a time it was found a good thing could come out of Nazareth, so, in these days, let us not shut our eyes to the possibility of finding some useful thing, if it be only one friend the more, which was "made in Germany."

### Everybody's Opinion.

[Correspondence on all subjects is invited, but we cannot in any way be responsible for the opinions expressed by our correspondents. No communication can be entertained if the name and address of the correspondent is not given, as a guarantee of good faith but not necessarily for publication, or unless one side of the paper only is written on.]

AN APPOINTMENT.

"A. F. SHEDDEN," the new superintendent at Dorking Workhouse Infirmary, writes: I have not been head maternity nurse at Hackney Union Infirmary ever since I left Birmingham. I came to Hackney last October, having held the post as sister at the Infirmary, Camberwell, for thirteen months. I have held several posts since 1892, in which year I left Birmingham.

[If Miss Shedden's name had appeared in "Burdett's Official Nursing Directory," the full details of her career could have been given.—ED. HOSPITAL.]

THE NURSES' CO-OPERATION.

"A NEW MEMBER" writes: It was with great pleasure I read the letter by "An Old Member of the Nurses' Co-operation" in your last issue. I have only been a member of the "Co." for a few months, but my experience has been such as to make me feel keenly the possibility of Miss Hughes giving up her position as lady superintendent. I feel, like "A Grieved Co. Nurse" that "Miss Hughes will never know how much she is worth to us." It seems to me that the committee have, by their action, unintentionally given the nurses a good deal to be thankful for. Instead of going quietly on, and passively, though loyally, feeling confidence in and appreciating their matron, they have been roused to realise acutely how fully Miss Hughes has their affection as well as esteem. When the present anxiety is over, and we can rejoice in feeling that Miss Hughes is still at our head, the past troubles will, we must hope, have achieved much; and cannot fail to form a strong bond of unity amongst the members of the Nurses' Co-operation.

ILL-CHOSEN NURSES.

"L. O. S." writes: Seeing from time to time letters about the kind of nurses that have been and are being sent out by public bodies to nurse in other countries, please allow me, as a sister of a great number of years' experience, to make a few remarks. It is not always the three years' trained and most thorough nurse that gets a matron's good recommendation. Why? Because matron does not like her personally, so therefore either does not give her a reference or perhaps may give an indifferent one. But the nurse who is smooth-tongued and easily led, no matter if she is untidy and slipshod in all her work, secures the passport to success, if she strokes sister down all right. No wonder at the public remarks which are made. In my judgment the matron's testimonial ought to be discarded altogether and the medical references alone carry weight. Already I can hear matron and sister protesting, but let each individually think whether she has hurt one or more nurses in her career. Certainly none but fully-trained women ought to be sent abroad.

NURSES IN INDIA.

"ONE WHO KNOWS" writes from India: Your issue of March 2nd has only just reached me, but if not too late in the day I should like to make a few comments on the letter from your correspondent "Placidity," who says she has had many years' experience of nursing in India, and then goes on to speak of a nurse who had to walk two miles twice daily to her hospital. If this is a sample of "Placidity's" experience, her time must have been spent in a hill station. Here in Madras the conditions of climate and temperature are such that for many months in the year no Englishwoman could walk two miles twice daily without being completely exhausted, and certainly quite unfit for a day's work in hospital. This condition of things applies to all Indian stations where there are large and important civil hospitals, or where there is any great demand for private nurses. If "Placidity's" letter has been called forth by the letters which appeared in the earlier issues of THE HOSPITAL for the current year on the subject of the treatment of the nurses in the General Hospital, Madras, I can assure her that English nurses in that institution work under conditions very different from those "Placidity" describes. True, their pay is not large, and their quarters are far less comfortable than they might be, but these drawbacks are not the trials which the numerous letters which appeared in your paper called attention to. Anyone reading the letters I refer to will see that the internal management is the cause of complaint. I have never heard of any English nurse leaving the Madras General Hospital on account of small pay, though I have known cases of nurses in other Indian hospitals resigning their appointments for this reason. "Placidity's" suggestion that English nurses come out to India and "pose as moneyed aristocrats" I consider a libel. I have, during my residence of many years in India, come in contact with English nurses in all three presidencies, and I have never known an English nurse to pose as a moneyed aristocrat. In my experience all English ladies working in India as nurses are treated as social equals by the whole English community. Regarding "Placidity's" remarks as to "thousands of poor whites" working in Indian cities on 150 rupees a month, she, of course, speaks at random. I do not know of any Indian city which contains thousands of poor Europeans with pay of 150 rupees or less; I, of course, except the military element, to which "Placidity's" remarks obviously do not apply. Anyone knowing the application of the term "poor white" in this country would hardly, I think, care to contemplate the idea of an Englishwoman, coming out to India as a hospital nurse, being brought under that category.

### Presentations.

PRESTON ROYAL INFIRMARY.—Miss Mitchell, who is leaving Preston Infirmary to take up another appointment, was presented last Saturday with a tea-service from the nursing staff, who had a musical evening in her honour.

### Appointments.

ATCHAM UNION WORKHOUSE INFIRMARY, NEAR SHREWSBURY.—Miss Elizabeth Barton has been appointed superintendent nurse. She was trained at Blackburn Union Infirmary, and has since been charge nurse in that institution, and nurse at Sheffield Royal Infirmary.

BARRINGTON HOSPITAL, LIMERICK.—Miss Kate Steen has been appointed matron. She was trained at the London Hospital, and has since been attached to Taunton Jubilee Nursing Institute, and matron of Kettering and District General Hospital.

BOOTLE INFECTIOUS DISEASES HOSPITAL.—Miss Margaret Paxton Kinnear has been appointed sister. She was trained at East Pilton Hospital, Leith, where she has since been assistant nurse.

CUMBERLAND INFIRMARY, CARLISLE.—Miss Elizabeth Hodges has been appointed matron. She was trained at Guy's Hospital, and for the last five years has held successively at that institution the posts of holiday sister, night sister, sister-in-charge of the out-patient department, sister of ward of 57 beds, and assistant matron.

EAST END MOTHERS' HOME.—Miss Alice Blomfield has been appointed lady superintendent. She was trained at the London Hospital, where she was afterwards appointed sister. She has since been sister at Queen Charlotte's Lying-in Hospital.

EAST PILTON HOSPITAL, LEITH, N.B.—Miss M. Taylor has been appointed sister. She was trained at Hull Royal Infirmary, and has since been sister of enteric wards at Monsall Hospital.

FLEMING COTTAGE HOSPITAL, ABERLOUR.—Miss Sidni Read has been appointed matron. She was trained at Glasgow Royal Infirmary, and has since been superintendent of the male ward in Devonshire Hospital, Buxton, matron of Salford Union Infirmary, Eccles, and matron of the Royal Infirmary, Dumfries.

KENDAL FEVER HOSPITAL.—Miss Agnes Altmont has been appointed matron. She was trained at the City Hospital, Edinburgh, and has since been staff nurse at Stockton-on-Tees Fever Hospital, and charge nurse and assistant matron at Yoker Fever Hospital, near Glasgow.

ROYAL BOSCOMBE HOSPITAL, BOURNEMOUTH.—Miss Lilian Bayley has been appointed matron. She was trained at the Seamen's Hospital, Greenwich, and the Hospital for Women, Soho Square.

ST. MARY'S HOSPITAL FOR SICK CHILDREN, PLAISTOW.—Miss Beatrice A. Cookes has been appointed matron. She was trained at Leeds General Infirmary, and has since been day sister at the Children's Hospital, Pendlebury, Manchester, night sister at the Metropolitan Hospital, Kingsland Road, and assistant matron at the Royal Infirmary, Preston.

STOCKTON-ON-TEES HOSPITAL.—Miss Mitchell has been appointed male ward and theatre sister. She was trained at the Royal Infirmary, Preston.

THROAT AND EAR HOSPITAL, GOLDEN SQUARE, LONDON.—Miss Emily Collins has been appointed night sister. She was trained at the Seamen's Hospital, Greenwich, and the Hospital for Women, Soho Square.

TODMORDEN WORKHOUSE HOSPITAL.—Miss Hannah Elizabeth Proudman has been appointed nurse in charge. She was trained at Salford Union Infirmary, and has since been charge nurse at the Hospital for the Isolation of Phthisis, County Asylum, Rainhill, night superintendent at King's Norton Union Infirmary, and charge nurse at Abbey Parish Hospital, Paisley. Miss Proudman holds the L.O.S. certificate.

ULSTER HOSPITAL FOR CHILDREN AND WOMEN, BELFAST.—Miss Elizabeth S. Tate has been appointed matron. She was trained at Liverpool Royal Infirmary, and has since been sister in charge at Birkenhead Union Infirmary, head midwife at the Victoria Nursing House, Cheltenham, and superintendent at Bethnal Green Infirmary.

### For Reading to the Sick.

LEAVE ALL TO GOD.

LEAVE all to God,
Forsaken one, and stay thy tears ;
For the Highest knows thy pain,
Sees thy sufferings and thy fears ;
Thou shalt not wait His help in vain,
Leave all to God.

Be still and trust !
For His strokes are strokes of love,
Thou must for thy profit bear ;
He thy filial fear would move,
Trust thy Father's loving care,
Be still and trust !

Know God is near !
Though thou think Him far away,
Though His mercy long have slept,
He will come and not delay,
When His child enough hath wept,
For God is near !

Oh teach Him not
When and how to hear thy prayers ;
Never doth our God forget,
He the cross who longest bears
Finds his sorrows' bounds are set,
Then teach Him not.

If thou love Him,
Walking truly in His ways,
Then no trouble, cross or death,
E'er shall silence faith and praise.

—*From* "*Lyra Germanica.*"

"Rest in the Lord ; wait patiently for Him." In Hebrew, "be silent to God, and let Him mould thee." Keep still, and he will mould thee to the right shape.—*Martin Luther.*

I have noticed that wherever there has been a faithful following of the Lord in a consecrated soul, several things have inevitably followed, sooner or later. Meekness and quietness of spirit become in time the characteristics of the daily life. A submissive acceptance of the will of God as it comes in the hourly events of each day; pliability in the hands of God to do or to suffer all the good pleasure of his will ; sweetness under provocation ; calmness in the midst of turmoil and bustle ; yieldingness to the wishes of others, and an insensibility to slights and affronts ; absence of worry or anxiety ; deliverance from care and fear ;—all these, and many similar graces, are invariably found to be the natural outward development of that inward life which is hid with Christ in God.—*H. W. S.*

In one way it is a specially hard struggle for you to resign yourself fully and freely. Life seems taken from you, whether you will or no. But face the fact that God means you to lose this life : that for you it must be all probation, and no mission ; all waiting, and no service. Accept your sentence ; make no claims on life ; lean first and foremost on Him who was "a sorrowful man, well acquainted with griefs." Then, and then only, can you enjoy safely the love and work which He sends to help you through the weary days. If your cheerfulness depends entirely on that outward stimulus, you are not making the right use of what God is lending you.

—*Lucy Soulsby.*

### Notes and Queries.

**The Editor is always willing to answer in this column, withou any fee, all reasonable questions, as soon as possible.**

**But the following rules must be carefully observed :—**

**1. Every communication must be accompanied by the name and address of the writer.**

**2. The question must always bear upon nursing, directly or indirectly.**

**If an answer is required by letter a fee of half-a-crown must be enclosed with the note containing the inquiry.**

*Deaf-imbecile.*

(114) I should feel obliged if you could tell me of a charitable institution which would receive a deaf and dumb imbecile child.—*Inquirer.*

There are no charitable institutions for deaf and dumb imbeciles, but the child might be eligible for the Darenth Schools for Imbecile Children (near Dartford, Kent) under the Metropolitan Asylums Board.

*Testimonials.*

(115) 1. To whom should I apply for testimonials on leaving a case ; to the medical attendant, to the friends of the patient, or to both ? 2. What notice and salary are due to the nurse on the death of a patient ? I have had charge of a permanent case, and have been paid monthly.—*W. E. G.*

1. Both. 2. In your case, the salary having been paid monthly, you are entitled to a month's notice and salary, unless otherwise arranged.

*Substitute.*

(116) I have had a slight accident and another nurse has been engaged to do my work. Who should pay my substitute—should I, or the family of my patient ?—*Nurse E.*

If the case is a permanent one, and you are engaged by the year, the family who retains your services provides for you in sickness. If, on the other hand, you are engaged by the day or week, your employers are able to pay you for the day or week and dismiss you. You should therefore have some definite arrangement as soon as possible. If, as we presume, your case is kept for you until you recover, you would either pay your substitute or waive your own fees.

*Child's Nurse.*

(117) Can you tell me the present address of the Norland Institute ?—it has been moved from Holland Park. Do the nurses there receive any hospital training ?—*E. A. B.*

Is there any institution where I could be taught the duties of children's nurse ? I want to be able to take charge of infants from the month, or of a delicate child permanently.—*Daphne.*

Would you tell me of an institution where I could get a lady nurse for two small children experienced in illness ?—*E. R. M.* (Liverpool).

1. The Norland Institute, 10 Pembridge Square, W.—2. *E. A. B.* No.—*Daphne.* See answer to *E. A. B.* *E. R. M.* Apply the Secretary of the Bureau for Employment of Educated Women, 8 Sandon Terrace, Liverpool, as well as to the Norland Institute.

*Training.*

(118) I am matron of a boys' college and would be glad if you could tell me where I could be trained in sick nursing without premium and with salary. I am thirty.—*A. J.*

I am thinking of taking up monthly nursing, but, before doing so, I have been advised to train in a large hospital for three years. I have been offered a vacancy in one of the principal Poor Law infirmaries in London. (2) Would this training do as well as the ordinary hospital course ? I am told that there is a lack of surgical training in a Poor Law institution. (3) Would this be a hindrance to my success as a monthly nurse ?—*Ina.*

After three years' training in an ophthalmic hospital, with previous experience in general nursing, should I be qualified to join a nurses' home for private work, or for district nursing ?—*Nurse Marion.*

Will you please tell me the best hospitals, and the terms for obtaining a good training in district work.—*A. W.*

I am anxious to train as a nurse. I should like to know if having been operated on for gastric ulcer will prevent my following that profession.—*F. J.*

Will you please tell me if a certificate of two years' training from the Cancer Hospital, and one year in a general hospital, recognised as a training school, will be accepted as equivalent to a three years' certificate ?—*A. C.*

Will you tell me if there is any hospital in London, where a married woman, living apart from her husband—through no fault of her own—could be trained : she is twenty-seven.—*Nurse Harrie.*

See the "Nursing Profession : How and Where to Train," for all particulars as to individual schools and terms for training.—*Ina.* You are well advised. Yes. (3) No.—*Nurse Marion.* Some ; not those of the highest standing.—*A. W.* Write to the General Superintendent, Queen Victoria's Jubilee Institute for Nurses, St. Katherine's Precincts, Regent's Park, N.W.—*F. J.* Not of necessity, but it would probably prevent your being accepted at a good training school—*A. C.* It would be recognised by some institutions, but not by all. Write to the Local Government Board, Whitehall, S.W., to know if that authority would regard it as a sufficient qualification for superintendent nurse of a workhouse.—*Nurse Harrie.* Probably, if you have no children to support ; but you had better ascertain by applying to the various training schools, and fully stating the circumstances of the case.

**Standard Books of Reference.**

**"The Nursing Profession : How and Where to Train." 2s. net ; post free 2s. 4d.**

"Burdett's Official Nursing Directory." 3s. net. ; post free, 3s. 4d.

"Burdett's Hospitals and Charities." 5s.

**"The Nurses' Dictionary of Medical Terms." 2s.**

**"Burdett's Series of Nursing Text-Books." 1s. each.**

**"A Handbook for Nurses." (Illustrated.) 5s.**

**"Nursing : Its Theory and Practice." New Edition. 3s. 6d.**

**"Helps in Sickness and to Health." Fifteenth Thousand. 5s.**

**"The Physiological Feeding of Infants." 1s.**

**"The Physiological Nursery Chart." 1s. ; post free, 1s. 3d.**

**"Hospital Expenditure : The Commissariat." 2s. 6d.**

**All these are published by THE SCIENTIFIC PRESS, Ltd., and may be obtained through any bookseller or direct from the publishers, 28 and 29 Southampton Street, London, W.C.**

